# A Hybrid Sender- and Receiver-Initiated Protocol Scheme in Underwater Acoustic Sensor Networks

**DOI:** 10.3390/s151128052

**Published:** 2015-11-05

**Authors:** Jae-Won Lee, Ho-Shin Cho

**Affiliations:** 1 Underwater Communication/Detection Research Center, Kyungpook National University, Daegu 702-701, Korea; E-Mail: jwlee@ee.knu.ac.kr; 2 College of IT Engineering, Kyungpook National University, Daegu 702-701, Korea

**Keywords:** handshake-based MAC protocol, handshake-sharing, hidden-node problem, long propagation delay, receiver-initiated protocol, sender-initiated protocol, sensor networks, spatial unfairness problem, underwater acoustics

## Abstract

In this paper, we propose a method for sharing the handshakes of control packets among multiple nodes, which we call a hybrid sender- and receiver-initiated (HSR) protocol scheme. Handshake-sharing can be achieved by inviting neighbors to join the current handshake and by allowing them to send their data packets without requiring extra handshakes. Thus, HSR can reduce the signaling overhead involved in control packet exchanges during handshakes, as well as resolve the spatial unfairness problem between nodes. From an operational perspective, HSR resembles the well-known handshake-sharing scheme referred to as the medium access control (MAC) protocol using reverse opportunistic packet appending (ROPA). However, in ROPA the waiting time is not controllable for the receiver’s neighbors and thus unexpected collisions may occur at the receiver due to hidden neighbors, whereas the proposed scheme allows all nodes to avoid hidden-node-induced collisions according to an elaborately calculated waiting time. Our computer simulations demonstrated that HSR outperforms ROPA with respect to both the throughput and delay by around 9.65% and 11.36%, respectively.

## 1. Introduction

Underwater acoustic sensor networks (UWSNs) comprise variable numbers of sensor nodes, which are deployed to perform a variety range of applications, such as oceanic research, oil spill monitoring, submarine detection, offshore exploration, and assisted navigation [1,2]. To communicate between nodes, UWSNs typically employ acoustic signals, which have poor channel conditions compared with terrestrial radio signals. In particular, the speed of sound under water is about 1500 m/s, which is lower than the 3 × 10^8^ m/s propagation speed of a radio signal, resulting in significantly longer propagation delays. In addition, the underwater acoustic channel is only capable of low data transmission rates due to its extremely limited bandwidth. Consequently, the medium access control (MAC) protocols designed for terrestrial radio channels with high data rates and negligible propagation delays may not work correctly in the underwater channel [3]. Therefore, it is essential to design a new MAC protocol to adapt to the poor underwater channel conditions.

Current research into underwater MAC protocols has focused mainly on handshake-based protocols, which are known to work properly during long-distance delivery via multi-hop relaying [4,5]. The multiple-access collision avoidance (MACA) protocol is a popular terrestrial handshake-based MAC protocol, which uses the request-to-send (RTS)/clear-to-send (CTS) handshake to reserve the shared channel [6]. In MACA, prior to data transmission the sender and receiver exchange RTS and CTS between the sender and receiver. Thus, any neighbors that overhear a control packet can delay their transmission to avoid possible collisions, which is called as a hidden-node problem. In order to apply MACA in an underwater environment, MACA for underwater (MACA-U) was proposed by revising the state transition rules to consider the long propagation delay [7]. However, the simple RTS/CTS exchange fails to fully address the hidden-node problem due to the long propagation delay in the underwater acoustic channel [8]. In addition, the performance of MACA-U is severely constrained by the long propagation delay because of the increased time required for control packet exchanges. Moreover, the long propagation delay also causes a spatial unfairness problem where the channel always becomes clear earlier at nodes closer to the receiver, and thus nodes located far from the receiver might never capture the channel.

Most conventional protocols have improved the channel utilization by sending multiple packets at once in a packet-train form [9,10,11]. As another approach for channel utilization improvement, a MAC protocol using reverse opportunistic packet appending (ROPA) has permitted multiple nodes to participate in a common handshake [12], which we name “handshake-sharing” in this paper. In general, the handshake-sharing approach is known to be more efficient than the packet-train methods, because the packet-train is only originated from a single sender. In ROPA, an initiating sender polls its neighbors to join the handshake using RTS, which may cause hidden-node collisions at the receiver-side. Such collisions may severely degrade the performance in terms of throughput and latency. Thus, as a solution for the hidden-node collisions, conventional protocols have inserted a specific amount of waiting time into the schedules of both the sender and receiver, but it may still cause additional latency [13,14,15]. In this paper, we propose a new handshake-sharing approach where a receiver polls its neighbors to join the handshake using CTS. Such a receiver-initiated polling not only improves channel utilization, but also addresses the hidden-node problem.

Based on these observations, we propose an underwater MAC protocol called a hybrid sender- and receiver-initiated (HSR) protocol scheme, which addresses the channel utilization issue and the hidden-node problem. First, to overcome the channel utilization problem, HSR permits multiple nodes to cut into an ongoing communication procedure that involves a handshake between a given sender and a receiver, thereby allowing data packets to be sent without additional handshakes. The method for sharing an ongoing handshaking is similar to that of ROPA. However, in contrast to ROPA, the receiver is the same for both the sender and neighbors in HSR, and thus the transmission schedules of all the nodes can be controlled by the same receiver to avoid the hidden-node problem. HSR combines both sender- and receiver-initiated approaches from the aspect of who initiates communication. The sender begins a handshake by transmitting an RTS to a receiver as part of the typical sender-initiated communication. On the other hand, the receiver invites neighbors to participate in the ongoing communication using three-way signaling via polling/request/grant as part of the receiver-initiated communication [11]. In addition, this participatory process gives neighbors the opportunity to transmit their data packets evenly, which also can address the spatial unfairness problem.

The remainder of this paper is organized as follows: we first review the relevant literature in Section 2. Section 3 presents the problem statements. In Section 4, we explain the proposed protocol design, including our system description. In Section 5, we present our simulation results and discuss them in detail. Finally, we give our conclusions in Section 6.

## 2. Related Work

Considerable research efforts have been made to overcome the effects of long propagation delay, such as low channel utilization, the hidden-node problem, and the spatial unfairness problem, which are also the main concerns of HSR. First, to improve channel utilization, a MACA-based MAC protocol with a packet-train to multiple neighbors (MACA-MN) was proposed wherein multiple packets could be sent to multiple neighbors in every handshake round [9]. Moreover, MACA-U with packet trains (MACA-UPT) was introduced [10] to enhance channel utilization by allowing a sender to transmit a train of data packets for each handshake. Another method for improving channel utilization was presented [8], which is called the adaptive propagation-delay-tolerant collision-avoidance protocol (APCAP). This protocol allows a transmitting node to perform other actions while waiting for the CTS to return, called MAC level pipelining. Nevertheless, APCAP has two constraints: the complexity of MAC level pipelining and time synchronization. Recently, a bidirectional concurrent MAC (BiC-MAC) protocol was proposed, where a sender-receiver pair can exchange bidirectional data transmissions in each handshake simultaneously, which results in channel utilization improvement [10]; however, a bidirectional data packet exchange only occurs when the receiver has data packets destined for the sender. If the receiver does not have any data packets to return, BiC-MAC behaves in a similar manner to MACA-UPT.

In a second attempt to overcome the hidden-node problem, so-called slotted floor acquisition multiple access (Slotted-FAMA) was proposed, which combines carrier sensing and RTS/CTS handshake mechanisms [13]. Slotted-FAMA exploits time slotting to prevent the hidden-node collisions by aligning all of the packet transmissions into slots, but which requires an excessive length of time slot resulting in a low throughput performance. Unlike Slotted-FAMA, the distance-aware collision avoidance protocol (DACAP) [14] is a non-synchronized protocol that minimizes the duration of handshake by taking advantage of the receiver’s tolerance to interference when the two nodes are closer than the maximum transmission range. Moreover, DACAP waits for a certain amount of time before data transmission to avoid possible hidden-node collisions. This waiting time is determined based on a trade-off between throughput and collision avoidance.

Finally, to address the spatial unfairness problem, the spatially fair MAC (SF-MAC) protocol was proposed, which also considers the hidden-node problem [15]. To avoid the hidden-node collisions, SF-MAC delays the CTS transmission after receiving the RTS. In addition, the receiver captures the RTSs of all contenders and determines the earliest RTS transmitter, thereby achieving a fair transmission. However, SF-MAC has a long and fixed RTS collection period and the receiver can only obtain a data packet from one of the potential senders, which severely degrades channel utilization. An efficient handshaking mechanism (EHM) was also proposed to solve the spatial unfairness problem [16]. In EHM, a receiver delays its reply to the RTS that arrives first for a specific amount of time. During this period, if any other RTS that departed earlier than the first-arrival RTS should arrive late due to the long distance, then the receiver also takes the late-arriving RTS into consideration when creating CTS so the long-distance node has an equal transmission opportunity. However, EHM does not consider the control packet collisions caused by hidden nodes, which may significantly decrease the overall network throughput.

## 3. Problem Statements

### 3.1. Hidden-Node Problem in UWSNs

Conventional handshake-based protocols attempt to reserve the channel by exchanging RTS/CTS control packets, which are probably overheard by neighbors. The neighbors are then aware that the channel will be reserved and they remain in the sleep mode by stopping any transmissions until the occupied channel is released. In this manner, any possible collisions caused by neighboring hidden nodes may be avoided.

**Figure 1:**
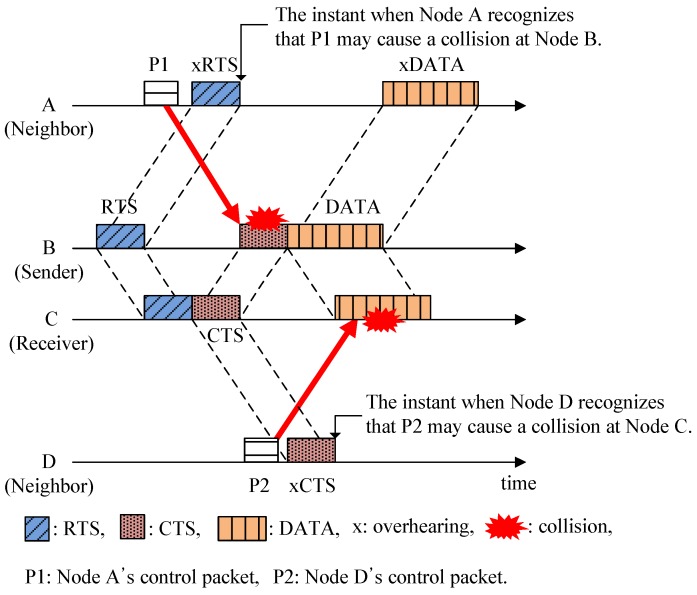
Illustration of the hidden-node problem in UWSNs.

However, in the underwater acoustic channel, a new type of hidden-node problem is introduced due to the long propagation delay. In Figure 1, nodes A and D, *i.e.*, the neighbors in this example, may detect a channel reservation too late by overhearing the RTS or CTS after completing the transmission of their control packets, P1 and P2. Thus, the early departure of packets without recognizing channel reservation may cause collisions at the sender (node B) and receiver (node C), as shown by the solid arrows. In this scenario, nodes A and D are hidden from nodes B and C. Therefore, unlike a terrestrial radio channel where a hidden node is located beyond the signal’s coverage so its existence is not recognized, a hidden node in an underwater acoustic channel may also occur due to the long propagation delay even when it is located within the region covered.

### 3.2. Spatial Unfairness Problem in UWSNs

The “first come, first served” method has been used as the most reasonable approach to ensure fairness among communicating nodes that share a medium, where the requests for the medium accessed by nodes are processed in the order of their arrival. However, due to the long propagation delay in the underwater acoustic channel, a request for channel reservation occurs earlier, but it may arrive later at a long-distance node compared with those nearby so it cannot be served first. This is called the spatial unfairness problem.

**Figure 2:**
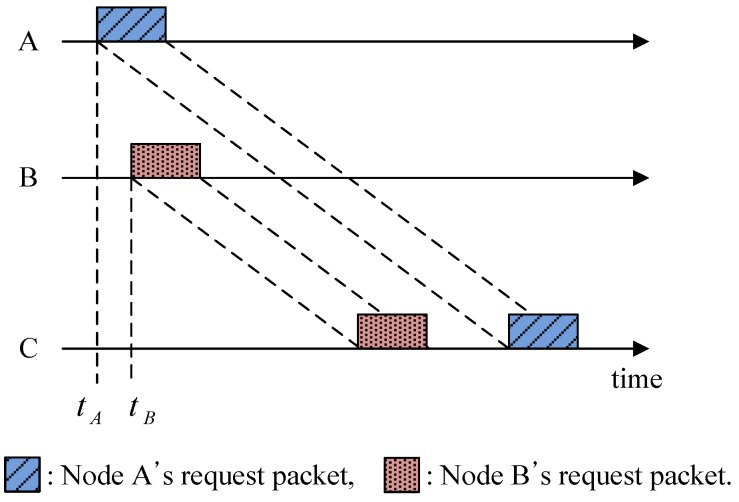
Illustration of the spatial unfairness problem in UWSNs.

In Figure 2, nodes A and B transmit a request packet to node C to reserve the channel. The starting time of node A *t_A_* is before *t_B_* for node B, but the request from node A arrives later than that from node B because node A is located farther than node B from node C. Furthermore, provided that node B has data to transmit, it can preoccupy the channel every time in advance of node A. This causes a severe unfairness problem.

## 4. Proposed HSR Protocol

### 4.1. System Description

In HSR, handshake-sharing can be achieved using multi-way polling/request/grant signaling between the receiver and its neighbors. A CTS plays an additional role in polling whether the neighbors have any data packets destined for the receiver. After receiving the CTS, the neighbors who want to participate in the current handshake (defined as “participants”) notify their participation by transmitting a control packet called a request-to-participate (RTP). After collecting RTPs, the receiver grants participation by broadcasting a control packet called a clear-to-participate (CTP), which contains the transmission schedules of the sender and participants to avoid collisions at the receiver. We refer to the sender and participants who are granted participation by the receiver as “granted participants”.

#### 4.1.1. Assumptions

A multi-hop acoustic network is considered where all of the nodes reach their respective destinations in a multi-hop manner using an omnidirectional and half-duplex underwater acoustic modem. We assume that each node acquires the inter-nodal propagation delay for one-hop distance neighbors through the network initialization stage by measuring the round-trip time or by exchanging some information with its neighboring nodes [17]. The nodes also maintain a list of neighbors to allow the establishment of a bi-directional communication link if necessary. A routing table is maintained in the nodes to facilitate multi-hop relay.

#### 4.1.2. Time Duration Parameters in HSR

In HSR, three parameters related to the time duration are defined, *i.e.*, Delay, Silence, and Waiting. First, Delay (denoted by *D^X^* (*i*)) is an interval during which a node *i* delays packet *X*’s transmission to avoid collisions. Silence is an interval during which nodes who overhear the control packets associated with channel reservation by others remain quiet and do not communicate. To express the length of the Silence, another time duration called Waiting is defined as an interval between the transmission of a control packet and the reception of the corresponding response, which could be a control packet or a data packet.

**Figure 3:**
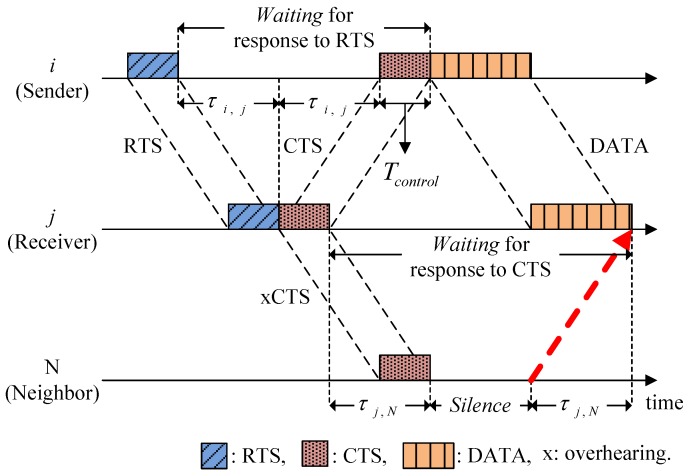
Illustration of the Waiting and Silence.

Figure 3 shows an example of a RTS/CTS handshaking procedure to illustrate how to obtain the Waiting and Silence. CTS is the response to RTS, so the Waiting of node *i* for response to RTS is given by:

(1) $$W^{RTS}\left(i\right)=2\tau_{i,j}+T_{control}$$

where *τ_i,j_* is the propagation delay between nodes *i* and *j*, and *T_control_* is the transmission time for the control packet, which is the same for all control packets. We denote *W^X^* (*i*) as the Waiting of node *i* to obtain a response to packet *X*. Similarly, since the data from node *i* correspond to the response to CTS of node *j*, then the Waiting of node *j* for response to CTS is given by:

(2) $$W^{CTS}\left(j\right)=2\tau_{i,j}+T_{DATA}$$

where *T_DATA_* is the transmission time of a data packet. The Waiting is specified in the associated control packets and it is overheard by neighbors so they can estimate how long they should remain in Silence in order to avoid collisions. In Figure 3, the values of *W^RTS^* (*i*) and *W^CTS^* (*j*) are specified in the RTS and CTS, respectively. After overhearing the CTS, the neighbor (node *N*) calculates the Silence by:

(3) $$S(N)=W^{CTS}\left(j\right)-2\tau_{j,N}$$

Similarly, all of the neighbors can calculate how long they must maintain the Silence. If a node in Silence overhears another control packet of neighbor, it checks and extends the Silence if necessary.

### 4.2. Fundamental Operation and Features

**Figure 4:**
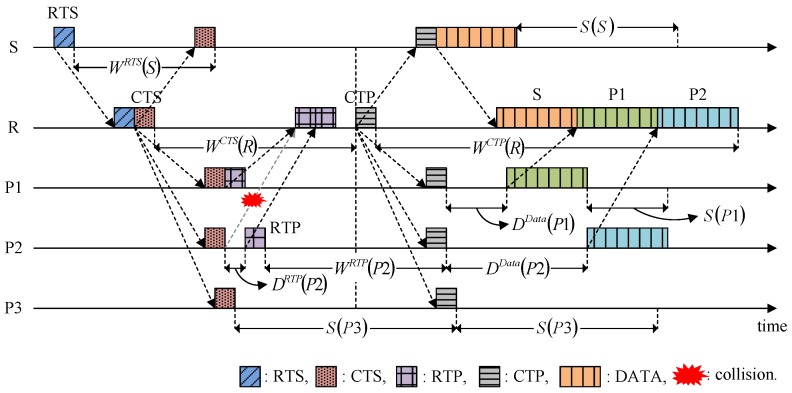
Operation of the HSR protocol.

Figure 4 illustrates the operation of the HSR protocol. Nodes S and R denote a sender and a receiver, respectively. It is assumed that nodes P1, P2, and P3 are neighbors of node R within one-hop distance. Node S starts the handshake by transmitting RTS to node R. Node R specifies the following information in the RTS: (1) the address of the destination; (2) the batch size of node S, *i.e.*, the number of data packets to be transmitted, *B_size,S_*; and (3) the Waiting of node S for response to RTS, *W^RTS^* (*S*).

After receiving the RTS, node R broadcasts a CTS, which is used to respond to the RTS but also to invite the neighbors (e.g., nodes P1, P2, and P3) to join the current handshake to send their data packets to the receiver R if they have any. After receiving the CTS, a neighbor with data packets destined for node R participates in the current handshake by transmitting an RTP to node R, and it then waits for a response. As shown in Figure 4, the RTPs from different participants might collide with each other, so node R computes the Delay for all of the participants’ RTP transmissions to ensure that collisions do not occur and it specifies these values in the CTS. Thus, the CTS contains two parameters: *W^CTS^* (*R*) and *D^RTP^* (*i*). The methods for calculating *W^X^* (*i*) and *D^X^* (*i*) are explained in Section 4.3. The RTP of participant *i* includes the same information as RTS: (1) address of the destination; (2) *B_size,i_*; (3) *W^RTP^* (*i*). On the other hand, a neighbor with no data packets destined for node R remains silent during the Silence (as in the case of node P3).

**Figure 5:**
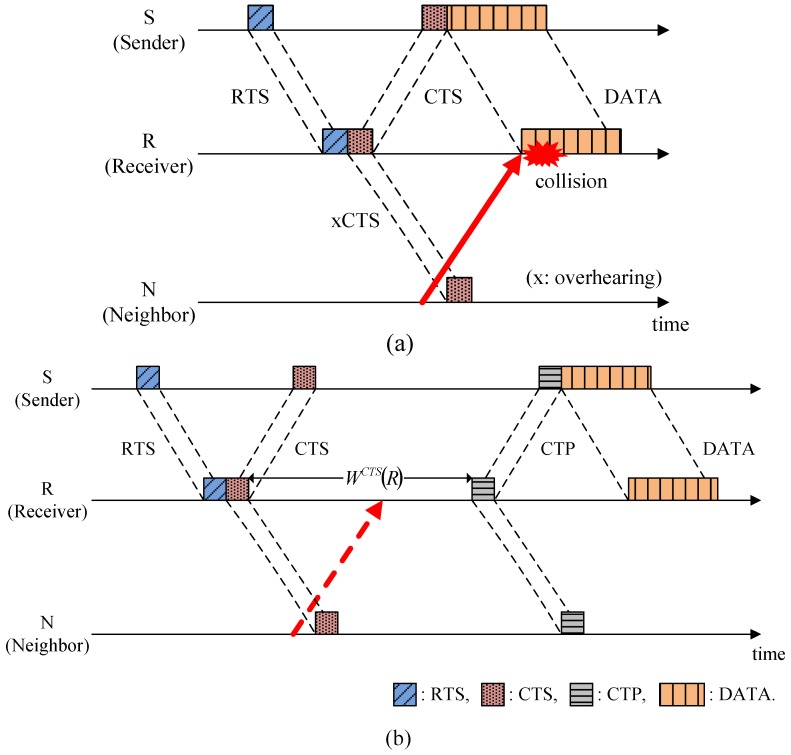
Solution to the hidden-node problem: (**a**) Conventional RTS/CTS handshaking protocol; and (**b**) HSR protocol.

After transmitting the CTS, node R collects RTPs during the Waiting, *W^CTS^* (*R*), and then broadcasts the CTP. In this case, *W^CTS^* (*R*) may induce slightly additional handshake negotiation times for a low offered load condition, but its contribution to the performance, such as collecting the neighbors’ notifications of participation and avoiding the collisions caused by hidden nodes, are high enough to ignore the demerit. As shown in Figure 5a, node N is hidden from node S and it may transmit a control packet (e.g., RTS) before overhearing CTS, which causes a collision at node R, as shown by the solid arrow. However, in HSR, *W^CTS^* (*R*) continues until the arrival of all of the control packets that departed from the neighbors earlier than the CTS was overheard, as shown by the dotted arrow in Figure 5b. This method allows the HSR to avoid possible collisions caused by hidden nodes.

**Figure 6:**
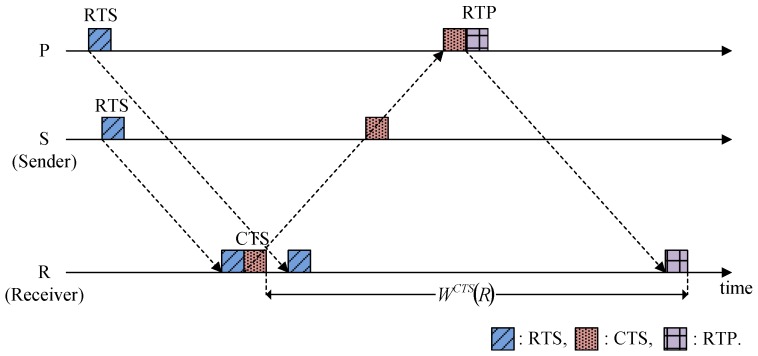
Solution to the spatial unfairness problem.

In addition, the HSR addresses the spatial unfairness problem caused by different inter-node distances and the long propagation delay, where it provides an additional opportunity to participate in the ongoing communication. For example, as shown in Figure 6, nodes S and P transmit their RTSs to node R. Because it is closer to node R, node S captures the channel even if it sends the RTS later than node P, thereby leading to the spatial unfairness problem. However, in HSR, node P still has a chance to share the channel by transmitting the RTP, as shown in Figure 6. Thus, all of the neighbors of the receiver have the same opportunity to capture the channel, regardless of their locations.

Back to Figure 4, after collecting all of the RTPs, node R calculates the data transmission times for the granted participants so the data packets arrive at the receiver sequentially in a similar manner to a packet-train [9]. The data transmission time for node *i* is translated into *D^Data^* (*i*) and sent with *W^CTP^* (*R*), where it is included in the CTS. After transmitting the CTP, node R waits for the data packets from the granted participants.

### 4.3. Calculating the Time Duration Parameters

#### 4.3.1. Delay for the RTP and Data Transmission

In HSR, two types of collisions can occur at a receiver: (1) RTP collisions and (2) data packet collisions. RTP collisions occur when more than two participants at similar distances from the receiver send an RTP immediately after receiving the CTS. Thus, in order to avoid RTP collisions, it is necessary to force all of the participants to delay their RTP transmissions by a specific amount of time, which is denoted by *D^RTP^*.

**Figure 7:**
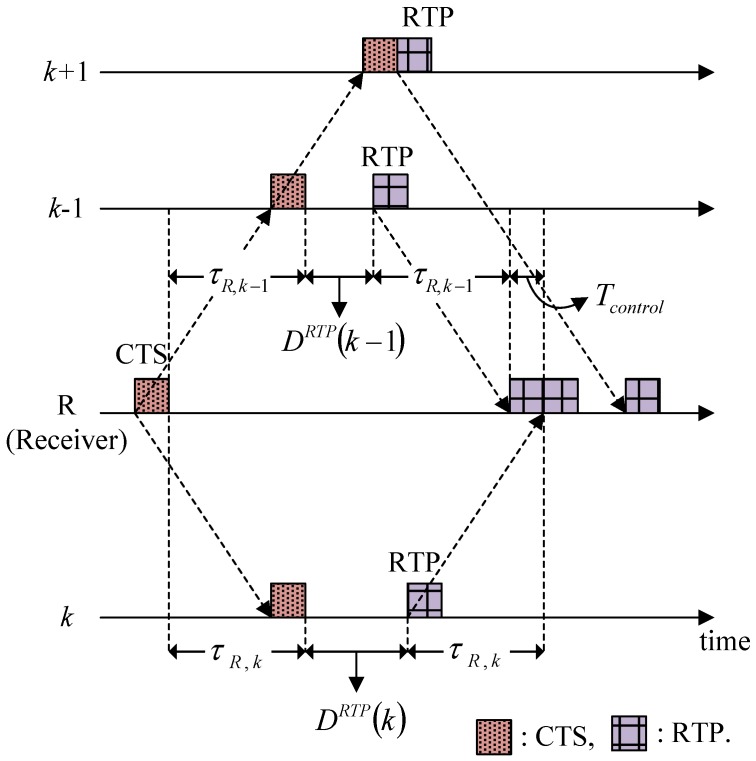
Determination of the Delay for RTP transmission.

Figure 7 shows an example of how to calculate the Delay for RTP transmission, where nodes (*k* − 1), *k,* and (*k* + 1) are participants, and their RTPs are assumed to arrive in the order named. The condition that node *k*’s RTP is scheduled to arrive after node (*k* − 1)’s RTP is given by:

(4) $$2\tau_{R,k-1}+T_{control}+D^{RTP}\left(k-1\right)\leq 2\tau_{R,k}+D^{RTP}\left(k\right)$$

Thus, the Delay for node *k*’s RTP transmission is chosen as the minimum value that satisfies the condition of Equation (4), as follows:

(5) $$D^{RTP}\left(k\right)=D^{RTP}\left(k-1\right)+T_{control}-2\left(\tau_{R,k}-\tau_{R,k-1}\right)$$

However, in the case where a Delay is not required, such as node (*k* + 1), Equation (5) yields a negative value. Thus, the general expression for the Delay for RTP transmission is given by:

(6) $$D^{RTP}\left(i\right)=\max\left(0,\;D^{RTP}\left(i-1\right)+T_{control}-2\left(\tau_{R,i}-\tau_{R,i-1}\right)\right)$$

*D^RTP^* (*i*) is calculated for all of the participants and written in the CTS by the receiver, and the participants then adjust the RTP transmission time accordingly.

**Figure 8:**
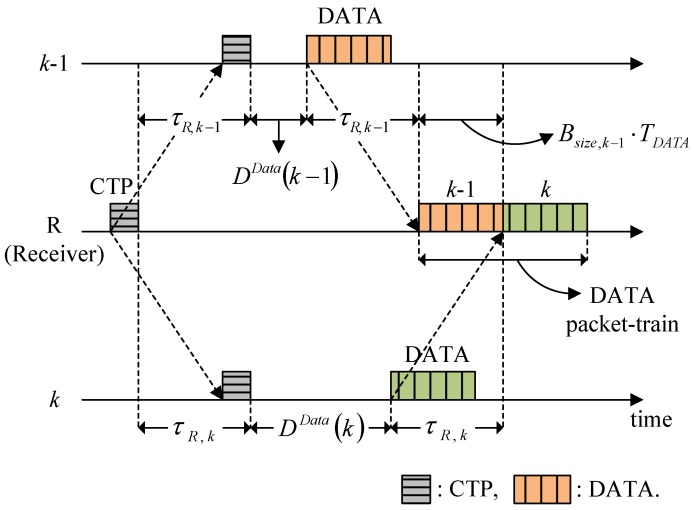
Determination of the Delay for data transmission.

Data packet collision is also avoided in a similar manner to RTP collision. After receiving the RTPs, the receiver then calculates the Delay for data transmission by the granted participants so the data packets arrive sequentially at the receiver in a packet-train form, as shown in Figure 8, where it is assumed that the data packets of node *k* arrive after those of node (*k − 1*). For this packet-train arrival process, the following relationship should be satisfied:

(7) $$2\tau_{R,k-1}+B_{size,k-1}\cdot T_{DATA}+D^{Data}\left(k-1\right)\leq 2\tau_{R,k}+D^{Data}\left(k\right)$$

Thus, from Equation (7), the Delay for node *k*’s data transmission is chosen as:

(8) $$D^{Data}\left(k\right)=D^{Data}\left(k-1\right)+B_{size,k-1}\cdot T_{DATA}-2\left(\tau_{R,k}-\tau_{R,k-1}\right)$$

and the general expression for granted participant *i* is:

(9) $$D^{Data}\left(i\right)=\max\left(0,\;D^{Data}\left(i-1\right)+B_{size,i-1}\cdot T_{DATA}-2\left(\tau_{R,i}-\tau_{R,i-1}\right)\right)$$

*D^Data^* (*i*) is carried by the CTP and the granted participants then determine their data transmission times accordingly.

#### 4.3.2. Waiting for Responses to RTS, CTS, RTP, and CTP

**Figure 9:**
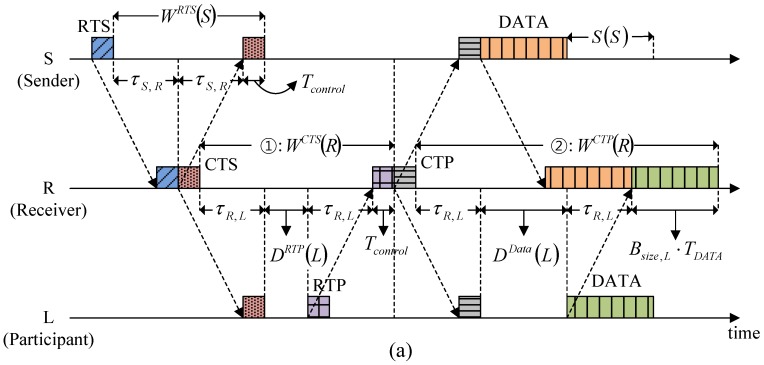

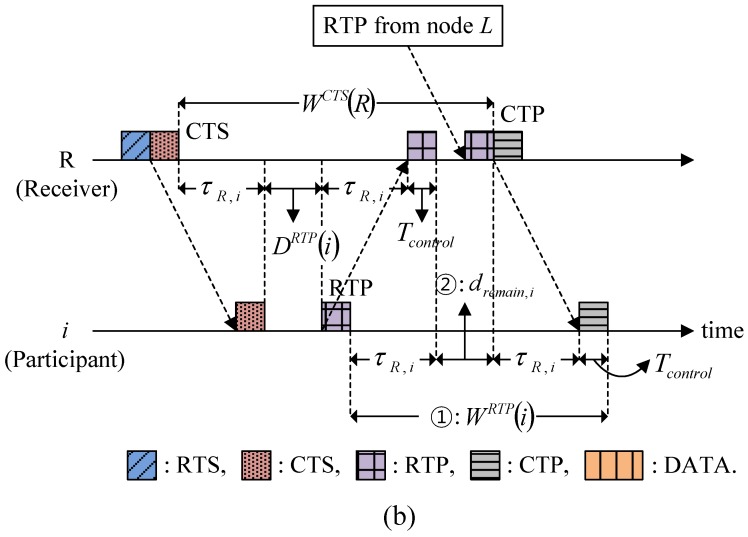
Determination of the Waiting for responses: (**a**) Associated with CTS and CTP; and (**b**) Associated with RTP.

The Waiting has a different value according to the type of control packet (*i.e.*, RTS, CTS, RTP, and CTP). The Waiting for response to RTS is given in Equation (1). As shown in Figure 9a, the Waiting for response to CTS (corresponding to ① in Figure 9a is given by:

(10) $$W^{CTS}\left(R\right)=2\tau_{R,L}+D^{RTP}\left(L\right)+T_{control}$$

where node *L* denotes the participant farthest from node R (receiver). The Waiting for response to CTP (corresponding to ② in Figure 9a, during which the data packet receptions are all completed as a response to CTP, is obtained by:

(11) $$W^{CTP}\left(R\right)=2\tau_{R,L}+D^{Data}\left(L\right)+B_{Size,L}\cdot T_{DATA}$$

where node *L* is the granted participant most distant from node R.

The Waiting of participant *i* for response to RTP (corresponding to ① in Figure 9b, during which CTP reception is completed as a response to RTP, is given by:

(12) $$W^{RTP}\left(i\right)=2\tau_{R,i}+T_{control}+d_{remain,i}$$

where *d_remain,i_* is the residual Waiting of node R for response to CTS at the time when participant *i*’s RTP is received (corresponding to ② in Figure 9b. Utilizing the values of *W^CTS^* (*R*) and *D^RTP^* (*i*), which are specified in CTS, participant *i* calculates *d_remain,i_* as:

(13) $$d_{remain,i}=W^{CTS}\left(R\right)-\left(2\tau_{R,i}+D^{RTP}\left(i\right)+T_{control}\right)$$

After substituting Equation (13) for Equation (12), the Waiting of participant *i* for response to RTP is given by:

(14) $$W^{RTP}\left(i\right)=W^{CTS}\left(R\right)-D^{RTP}\left(i\right)$$

## 5. Simulations and Results

### 5.1. Simulation Model

**Figure 10:**
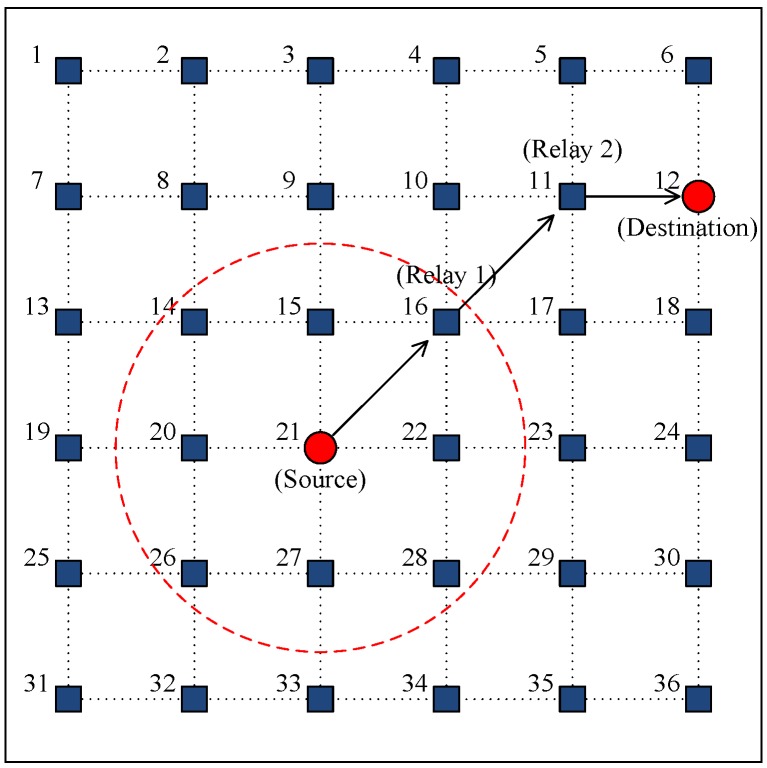
Network topology used in the simulations.

We developed a custom MATLAB network simulator and considered a multi-hop topology that has 36 static-nodes with a grid spacing of 1000 m as shown in Figure 10. To reflect more real situation, the location of node is randomly generated to deviate from a grid intersection point by 10% at maximum of the grid spacing in both vertical and horizontal directions. The transmission power of all of the nodes were assumed to be the same to cover 1.5 times the grid spacing, so the coverage of a node included eight neighbors, as denoted by the dotted circle in Figure 10.

Data packet generation for each node followed a Poisson distribution with parameter *λ_node_* (packets/s) and the destination of each packet was selected randomly with equal probability. In the case of multi-hop transmission, a static routing scheme was employed where every node used a preconfigured routing table [12]. We ignored any channel related packet losses due to noise and multi-path, which only occurred due to packet collisions. For the RTS trials, HSR employed the binary exponential back-off algorithm specified in the IEEE 802.11 standard [18]. Table 1 provides the system parameters and the values used in simulations. The transmission rate was referred to the LinkQuest acoustic modem [19].

**Table 1:** System parameters and values.

Parameter	Value
Transmission Rate	9600 bps
Size of Data Packet	1200 bits
Size of Control Packet	120 bits
Minimum Back-Off Counter	1
Maximum Back-Off Counter	64
Capacity of Buffer	300 packets
Speed of Acoustic Wave	1500 m/s

### 5.2. Simulation Results

We compared the HSR protocol with conventional handshake-based protocols, *i.e.*, MACA-U [7], MACA-UPT [10], and ROPA [12], with respect to the normalized throughput per node and end-to-end packet delay. The normalized throughput per node is defined by:

(15) $$\gamma =\frac{1}{N}\cdot \frac{\sum_{i=1}^{N}r_{i}\cdot B_{Data}}{t_{sim}}$$

where *r_i_* is the number of successfully received data packets at node *i* as a destination; *N* is the total number of nodes in the network; *B_Data_* is a data packet size in bits; and *t_sim_* is the total simulation duration. As another performance evaluation metric, the end-to-end packet delay is used, defined as the time spent from initial data generation at a source to successful reception at a destination. Let Ω be the set of successfully received data packets at a destination. The length of Ω is given by $N\left(\Omega \right)=\sum_{i=1}^{N}r_{i}$, and each element of Ω has a different end-to-end packet delay, $t_{delay,j},\;j=1,\;2,\;\cdots ,\;N\left(\Omega \right)$. Therefore, the average value of end-to-end packet delay is defined by:

(16) $$\bar{t_{delay}}=\frac{\sum_{j=1}^{N\left(\Omega \right)}t_{delay,j}}{N\left(\Omega \right)}$$

In terms of the channel occupancy priority, the three priority strategies of foreign-first, dominant-first, and oldest-first described in [20] were applied in order. Foreign-first gives priority to the relayed packets over newly generated packets. Dominant-first gives priority to a larger group of packets, which are commonly destined for a specific node. Oldest-first gives priority to the packets that have traveled a larger number of hops up to the current instant.

#### 5.2.1. Throughput and Delay Analysis

**Figure 11:**
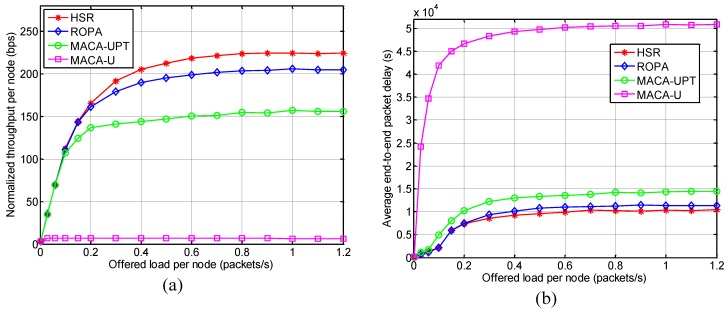
Performance comparison between HSR and other underwater MAC protocols: (**a**) Normalized throughput per node; and (**b**) Average end-to-end packet delay.

Figure 11 shows the normalized throughput per node and the average end-to-end packet delay performance for various offered loads per node (*λ_node_*). For simplicity, hereafter the terms “normalized throughput per node”, “average end-to-end delay”, and “offered load per node” are referred to as “throughput”, “delay”, and “offered load”, respectively. 

The results showed that MAC protocols with packet-train approach, such as HSR, ROPA, and MACA-UPT, significantly outperformed the non-packet-train MACA-U protocol in terms of both the throughput and delay, while achieving a stable saturation throughput and delay at high offered load ranges. This is because individual handshake for every single data packet is a very inefficient method, especially in conditions with a long propagation delay such as an underwater channel. Allowing multiple nodes to share an ongoing handshake, HSR and ROPA performed better than MACA-UPT where handshake only involved the sender and receiver.

**Figure 12:**
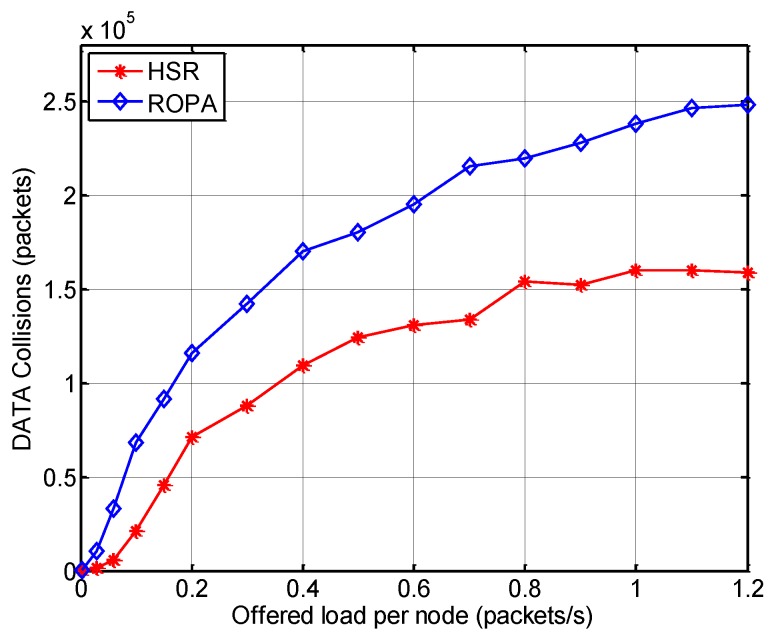
Number of data packet collisions with HSR and ROPA.

In the saturation region, a closer analysis of the results for HSR and ROPA showed that HSR performed better in terms of both throughput and delay by around 9.65% and 11.36%, respectively because HSR additionally addresses the hidden-node problem, as mentioned earlier. Figure 12 demonstrates that the number of data packet collisions was lower for HSR compared with that for ROPA and the difference increased with the offered load. The features of the aforementioned MAC protocols are summarized in Table 2.

**Table 2:** Comparison of the features of MAC protocols.

	Feature	Handshake-Sharing	Packet-Train Method	Solving the Hidden-Node Problem
MAC Protocol				
HSR		O	O	O
ROPA		O	O	X
MACA-UPT		X	O	X
MACA-U		X	X	X

O: used, X: not used.

**Figure 13:**
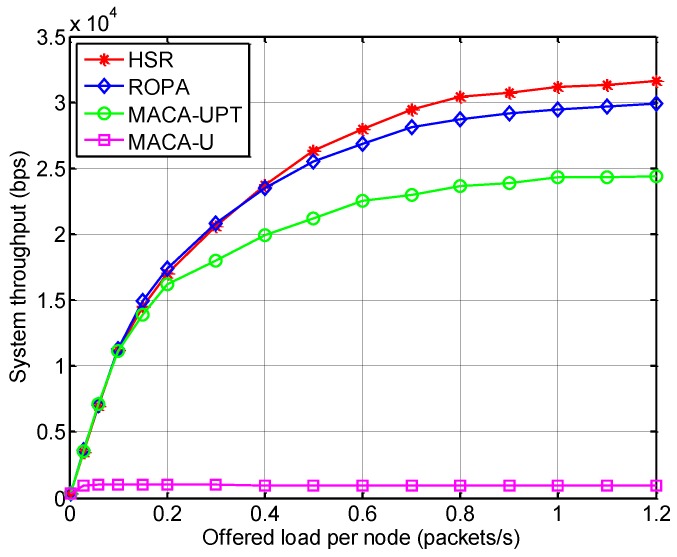
System throughput for HSR compared with other MAC protocols.

Figure 13 shows the system throughput for various offered loads, which is defined by:

(17) $$S=\frac{\sum_{i=1}^{N}s_{i}\cdot B_{Data}}{t_{sim}}$$

where *s_i_* is the total number of successfully received data packets at node *i*, regardless of whether they are relayed or destined. Alternatively, the system throughput can be interpreted as the single-hop performance of the MAC protocol. Thus, with similar reason as the case of throughput, HSR outperformed the other method in terms of the system throughput, which demonstrates that the features of HSR mentioned in Table 2 are also useful in single-hop networks.

#### 5.2.2. Analysis of Spatial Fairness

To obtain further insights into the performance of HSR, the spatial fairness is analyzed using Jain’s fairness index (FI), which is defined by [21]:

(18) $$FI=\frac{{\left(\sum_{i=1}^{N}x_{i}\right)}^{2}}{N\cdot \sum_{i=1}^{N}x_{i}^{2}}$$

where *x_i_* is the throughput of node *i*. The fairness index ranges from 1/N (worst case) to 1 (best case), and the maximum value can be obtained when all of the nodes have the same level of throughput.

**Figure 14:**
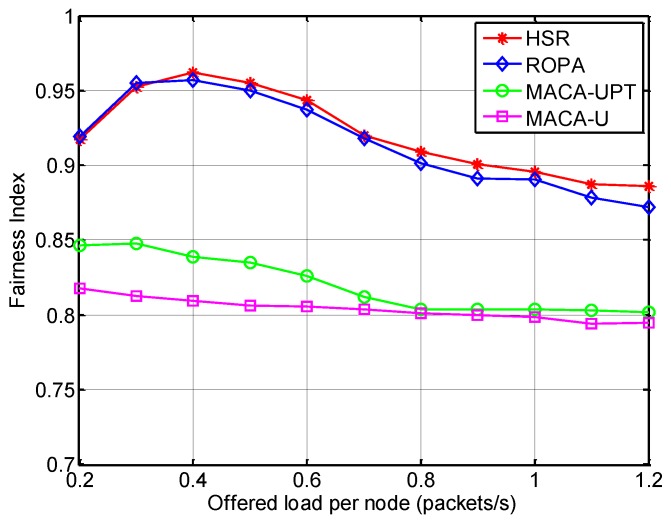
Fairness index for HSR compared with other MAC protocols.

Figure 14 shows that the fairness index was highest for HSR, which is because HSR gives all the neighbors of a receiver an equal opportunity to capture the channel regardless of their locations. ROPA also employs a handshake-sharing approach, but HSR offers a solution to the hidden-node problem and this helps to improve the fairness.

## 6. Conclusions

In this paper, we have considered the challenges posed by the long propagation delay in underwater acoustic channels when designing an underwater MAC protocol. Based on these considerations, we have proposed an underwater MAC protocol called the HSR, which employs a packet-train method to improve channel utilization as well as allowing multiple nodes to share an ongoing handshake between a sender and a receiver. This approach also reduces the latency, which is expected to be very high in long propagation delay environments such as those in underwater channels. In addition, HSR addresses the hidden-node problem, which occurs in a different manner compared with terrestrial networks, and this is the main cause of performance degradation in the underwater handshake-based MAC protocol. Addressing the hidden-node problem also improves the fairness between nodes. The results of computer simulations showed that HSR surpasses other popular underwater MAC protocols based on handshake or packet-train methods, such as MACA-U, MUAC-UPT, and ROPA, with respect to both the throughput and delay.
